# Management of Middle Hepatic Vein Injury during Laparoscopic Cholecystectomy: A Case Report

**DOI:** 10.1055/s-0040-1701695

**Published:** 2020-03-09

**Authors:** Juan Jose Santivañez, María Elena Velásquez, Manuel Cadena, Arturo Vergara

**Affiliations:** 1 Department of General Surgery, Hospital Universitario Fundación Santa Fe de Bogotá, Bogotá, Colombia; 2 Department of General Surgery, Universidad El Rosario, Bogotá, Colombia; 3 Department of Metabolic Support and Surgery for Intestinal Failure, Hospital Universitario Fundación Santa Fé de Bogotá, Bogotá, Colombia

**Keywords:** laparoscopic cholecystectomy, middle hepatic vein, hemorrhage

## Abstract

**Background** Cholecystectomy continues to be the first choice for the treatment of symptomatic cholelithiasis. Especially in patients with acute cholecystitis, a laparoscopic approach has become the standard treatment option. Intraoperative complications of laparoscopic cholecystectomy include: bile duct injury, organ damage, and bleeding due to vascular injury. Difficult hemorrhage during laparoscopic cholecystectomy occurs in 0.1 to 1.9% of all cases. Besides major vessel injuries, gallbladder bed vasculature is reported as a common injury site, mostly secondary to middle hepatic vein lesions.

**Case Presentation** We present a case report of a patient taken for a laparoscopic cholecystectomy. During the procedure, inadvertent middle hepatic vein injury occurs. Here we describe the management approach selected for this type of injury.

**Discussion** We recommend careful dissection during the final steps of a laparoscopic cholecystectomy. Following cystic duct and cystic artery ligation, surgeons often inappropriately relax through the last part of the dissection. During this final dissection, if care is not taken, small vascular structures can be missed and injured.

Cholelithiasis is a major public health problem in developed countries, affecting up to 20% of the population. 1 Cholelithiasis is responsible for 90 to 95% of cases of acute cholecystitis, and 2% of patients with nonsevere cholecystitis experience recurrence within 8 to 10 weeks. 2 Cholecystectomy is the first choice for the treatment of symptomatic cholelithiasis, especially for patients with acute cholecystitis. 3 Intraoperative complications of laparoscopic cholecystectomy include: bile duct and organ injury and bleeding due to vascular injury. Uncontrollable hemorrhage during laparoscopic cholecystectomy occurs in 0.1 to 1.9% of all cases, 4 leading to conversion to open surgery in up to 2% of all laparoscopic cholecystectomies. Furthermore, in 88% of these events, bleeding originates from the gallbladder bed. 5 The gallbladder bed is reported as a common vascular injury site, mostly secondary to trauma to the middle hepatic vein. 6 We present a case report of the management of a middle hepatic vein injury during laparoscopic cholecystectomy.

## Case Presentation

**Fig. 1:**
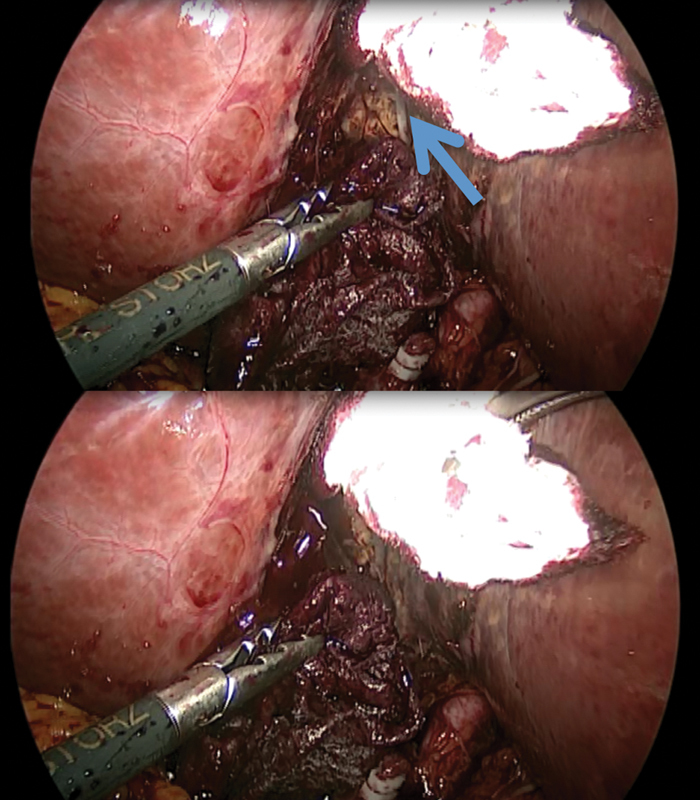
Dissection of the gallbladder bed during laparoscopy. A vascular structure was observed producing significant amount of blood (arrow on the upper image).

A 67-year-old male presented to our hospital after experiencing right upper quadrant abdominal pain for the last 2 weeks. Clinical history included treatment for follicular B lymphoma, stage IIA, and during his checkups a computed tomography (CT) scan of the abdomen was performed describing the presence of cholelithiasis. The patient was scheduled for an elective laparoscopic gallbladder removal on an outpatient basis. The day of the surgery, the patient was in good general health with normal vital signs. Abdominal examination revealed mild abdominal pain without signs of an acute abdomen. During the procedure, the hepatocystic triangle was dissected without complications. During dissection of the gallbladder from the cystic plate, a major venous hemorrhage erupted. Direct compression and electrocautery were not successful in controlling the bleeding. Fig. 1 . Due to continuous bleeding, hemodynamic changes and the inability to control bleeding with laparoscopy, the laparoscopy was aborted and a supraumbilical laparotomy incision was performed. The bleeding was controlled with ligation of the vessel and the peritonization of the gallbladder bed. The patient had a successful recovery and was discharged on postoperative day 3. On outpatient follow-up, the patient remained asymptomatic.

## Discussion

**Fig. 2:**
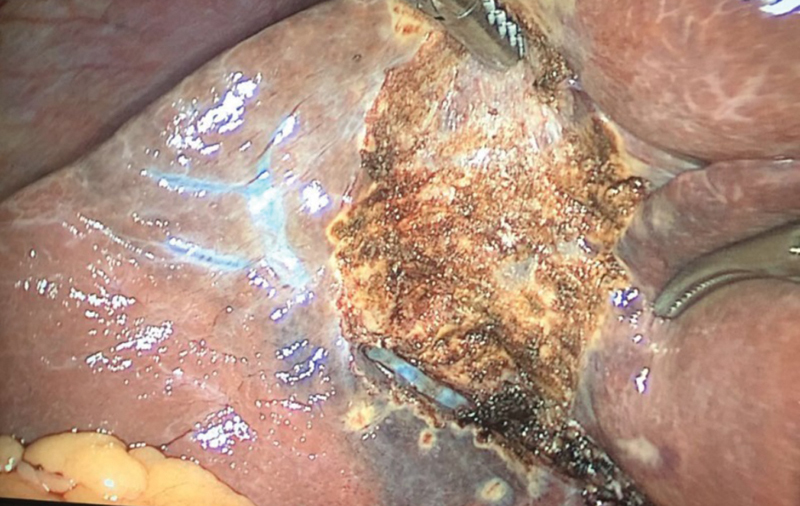
Middle hepatic vein seen in laparoscopy.

Laparoscopic cholecystectomy has been established as the gold standard for the treatment of gallstone disease, but it can be associated with significant morbidity and mortality. 7 Bleeding complications are an important cause of mortality, especially when facing major bleeding during a laparoscopic procedure where the bleeding control can be technically challenging. Between 10 and 15% of patients will display a large branch of the middle hepatic vein adherent to the gallbladder bed, presenting an increased risk of vein injury during cholecystectomy. Excluding major vessels, bleeding can originate from the gallbladder bed itself, and the middle hepatic vein has been described to be a cause of uncontrollable bleeding. 4 8 Misawa et al reported that the branch of the middle hepatic vein was completely adherent to the gallbladder bed in 5 of the 50 patients, and in one patient the diameter of the branch was 3.5 mm. In three patients, branch diameters were 3.0 to 3.8 mm traversed as close as 1.0 mm from the gallbladder bed. 6 The literature describes varies reflections on how to face this possible complication. Some proposed strategies include delayed cholecystectomy and using low energy cauterization. Other suggestions include a screening method to determine the middle hepatic vein distance from the gallbladder bed before laparoscopy. 6 As reported in this case, middle hepatic vein injury is an uncommon yet eventful situation. It is critical that the surgeon keeps in mind this anatomy, especially during the final steps of gallbladder dissection from the plate during laparoscopic cholecystectomy Fig. 2 .
